# Long-term nutritional status after total gastrectomy was comparable to proximal gastrectomy but with much less reflux esophagitis and anastomotic stenosis

**DOI:** 10.3389/fonc.2022.973902

**Published:** 2022-10-25

**Authors:** Shikang Ding, Xiaohao Zheng, Shenghui Wang, Ming Wu, Yunzi Wu, Chunyang Sun, Lin Yang, Liyan Xue, Bingzhi Wang, Chengfeng Wang, Yibin Xie

**Affiliations:** ^1^ Department of Pancreatic and Gastric Surgery, National Cancer Center/National Clinical Research Center for Cancer/Cancer Hospital, Chinese Academy of Medical Sciences and Peking Union Medical College, Beijing, China; ^2^ Department of General Surgery, Civil Aviation General Hospital, Beijing, China; ^3^ Department of Gastrointestinal Surgery, Yun Cheng Center Hospital, Yuncheng, China; ^4^ Department of General Surgery, The Central Hospital of Jia Mu Si City, Jiamusi, China; ^5^ Department of Medical Oncology, National Cancer Center/National Clinical Research Center for Cancer/Cancer Hospital, Chinese Academy of Medical Sciences and Peking Union Medical College, Beijing, China; ^6^ Department of Pathology, National Cancer Center/National Clinical Research Center for Cancer/Cancer Hospital, Chinese Academy of Medical Sciences and Peking Union Medical College, Beijing, China; ^7^ State Key Lab of Molecular Oncology, National Cancer Center/National Clinical Research Center for Cancer/Cancer Hospital, Chinese Academy of Medical Sciences and Peking Union Medical College, Beijing, China; ^8^ Department of Pancreatic and Gastric Surgery, National Cancer Center/National Clinical Research Center for Cancer/Hebei Cancer Hospital, Chinese Academy of Medical Sciences, Langfang, China

**Keywords:** proximal gastrectomy (PG), total gastrectomy (tg), nutritional status, reflux esophagitis, anastomotic stenosis

## Abstract

**Aim:**

To compare the long-term nutritional status, reflux esophagitis and anastomotic stenosis, between total gastrectomy (TG) and proximal gastrectomy (PG).

**Methods:**

Patients who underwent PG or TG in this single institution between January 2014 and December 2016 were included in this study. The inclusion and exclusion criteria were defined. One-to-one propensity score matching (PSM) by the demographic and pathological characteristics was performed to compare the long-term outcomes between the two groups. The primary endpoint was long-term nutritional status, and the second endpoints were reflux esophagitis and anastomotic stenosis. Long-term nutritional status was valued by percentage of body mass index (%BMI), body weight, and blood test including total protein, prealbumin, hemoglobin and total leukocytes.

**Results:**

Totally 460 patients received PG or TG in our institution for the treatment between January 2014 and December 2016 and according to the inclusion and exclusion criteria 226 cases were included in this study finally. There was no significant difference as to nutritional status in the end of first 5 years after PG or TG. While reflux esophagitis and anastomotic stenosis were significantly higher in the PG group than in the TG group (54.4% versus 26.8%, p < 0.001; 14.9% versus 4.5%, p=0.015; respectively). Overall survival rates were similar between the two groups after PSM (5-year survival rates: 65.4% versus 61.5% in the PG and TG groups, respectively; p = 0.54). The rate of carcinoma of remnant stomach after PG was 3.5% in this group of patients.

**Conclusions:**

TG should be more aggressively recommended for the similar nutritional status, significantly lower reflux esophagitis and anastomotic stenosis, and free of carcinoma of remnant stomach compared with PG.

## Introduction

Gastric cancer remains the fifth most frequently diagnosed cancer and the third most common cause of cancer-related death worldwide, especially in Eastern Asia, Eastern Europe, and South America (1–3). The epidemiological characteristics of gastric cancer have changed over the last several decades. That’s the incidence of esophageal-gastric junction (EGJ) cancer increased while the overall incidence of gastric cancer decreased in both Western and Asian countries (4, 5). Surgical resection remained the first choice of therapy for the EGJ cancer and other multimodal therapy strategies were combined if necessary, such as chemotherapy, radiotherapy, targeted therapy, and immunotherapy (6). Although both total gastrectomy (TG) and proximal gastrectomy (PG) were mostly used methods for EGJ cancer, the organ-preserved gastrectomy was strongly recommended recently, and more and more PG was performed in EGJ cancer with advanced stage (7, 8). However, according to more and more clinical data, TG didn’t inevitably mean a poor nutritional status, while more reflux esophagitis and anastomotic stenosis would occur after PG.

Many studies had been conducted to compare the nutritional status of patients underwent PG or TG for the early EGJ cancer, and the results were controvesial (9–12). In a retrospective study, the hematologic and nutritional status of 38 PG and 42 TG at the time of 24 months after surgery were compared. No significant differences in changes in hemoglobin (P=0.250), cumulative incidence of iron deficiency anemia (P=0.971), cumulative incidence of vitamin B12 deficiency (P=0.087), BMI changes from baseline (P=0.591), or nutritional outcomes were observed. It seemed as the time prolong, the nutritional status of PG and TG tended to be similar. Besides, a multicenter prospective trial showed that the PG group better than the TG group.

Furthermore, the hotly debated nutrition state was only one aspect to be concerned after PG or TG, life quality was also very important to those long-time survivors. Reflux esophagitis, anastomotic stenosis and carcinoma of remnant stomach made many patients suffered much. Many studies reported that the reflux symptoms after PG was prevalent, and one of which found that the reflux rate can be as high as 48% within 5 years, and if assessed by the LA grade or Visick grade classification reflux esophagitis was much more severe in the PG group than the TG group (11, 13, 14). Meanwhile, it’s well known that the rate of anastomotic stenosis in PG group was higher than TG group.

However, these studies were limited to early gastric cancer, and the long-term nutritional status, reflux esophagitis and anastomotic stenosis of those patients who survived more than 5 years were rarely studied across all gastric cancer stages. In the present study, we tried to make clear this question by the data of our single institution.

## Method

### Patient

In total, 2890 gastric cancer patients underwent gastrectomy at the Cancer Hospital Chinese Academy of Medical Sciences, from January 2014 to December 2016, and 460 patients who received PG or TG were included in this study. The inclusion criteria for this study were as follows: proximal or total gastrectomy; pathologically diagnosed as primary gastric adenocarcinoma; no other malignant tumor history; regular follow-up and received blood test in this institution every 3 months in the first 2 years after surgery and every 6 months in the next 3 years. The excluding criteria: Those patients who refused to answer the questionnaire were excluded.

### Treatment

Treatment methods for all patients were decided by a multidisciplinary treatment (MDT) group including at least radiologists, pathologists, medical oncologists, and surgeons, according to the preoperative assessment of contrast-enhanced chest–abdomen–pelvis computed tomography, upper gastrointestinal endoscopy and other examinations. Patients with clinical TNM stage III received 2-4 cycle neoadjuvant chemotherapy with SOX regimen. The criteria for choosing type of surgery were mainly based on the “Japanese gastric cancer treatment guidelines (ver. 4)”. According to the guidelines, the selection criteria for EGJ cancer surgery are as follows: PG: Tumor size ≤ 4cm, cT1 and lymph node metastasis (No. 1, 2, 3, 7); cT2 or above and lymph node metastasis (No. 1, 2, 3, 7, 8, 9, 11p, 11d, 19, 20). TG: Clinically node-positive (cN+) or T2–T4a tumors. PG was performed in a resection region of standard D2 lymphadenectomy except No. 5 and 6 group lymph nodes and the digestive tract reconstruction was accomplished by gastro-esophagectomy. TG was performed in a resection region of standard D2 lymphadenectomy, and the reconstruction of digestive tract was achieved by Rox-en-Y esophagojejunostomy. Additionally, all patients with TNM stage II-III were recommended for postoperative adjuvant chemotherapy administrated by the physician.

### PSM analysis

PSM analysis was conducted using a logistic regression model and the following covariates: age, sex, body mass index (BMI), tumor size, histological type, pathological TNM stage. Among them, sex, age, BMI, and tumor size were nearly matched, and the P value > 0.2 was set as nearest match; pathological TNM staging was set as a binary variable, which were I-II and III-IV, respectively. Finally, the Borranmm type and the pathological TNM staging were matched exactly one-to-one. The clinicopathological characteristics, short-term and long-term complications and long-term life status including long-term nutritional outcomes, recurrence and survival, were compared between the PG and TG groups after PSM.

### Study endpoint

The primary endpoint was the long-term nutritional status, and the second endpoints were reflux esophagitis and anastomotic stenosis. Long-term nutritional status was valued by percentage of body mass index (%BMI), body weight, and blood test including total protein, prealbumin, hemoglobin and total leukocytes.

### Data collection

Each of the follow-up data including BMI, weight and hematological indicators at each time of follow-up were calculated based on the preoperative data and took the percentage value into analysis. Postoperative complications were graded according to the Clavien–Dindo (CD) classification of surgical complications, and those events which were grade II or higher were included into analysis (15). Reflux symptoms were evaluated using the Visick grade classification, and reflux esophagitis was confirmed by endoscopic examination at the time of follow-up and was assessed using the Los Angeles (LA) classification (16, 17). Severe esophagitis was defined as grade C or D judged by the LA classification.

### Statistical analysis

R version 4.1 software (www.r-project.org ) was used for all statistical analyses. Chi-square tests for categorical variables and the Student’s test for unpaired data for continuous variables were performed to compare clinicopathological characteristics between the two groups. A p value of <0.05 was regarded as significant. Survival rates were calculated by the Kaplan–Meier method. In addition, the SVA package function Combat was used to remove the batch effect of laboratory items if the detection method changed over the years.

## Results

### Patient characteristics and surgical outcomes

**Figure 1:**
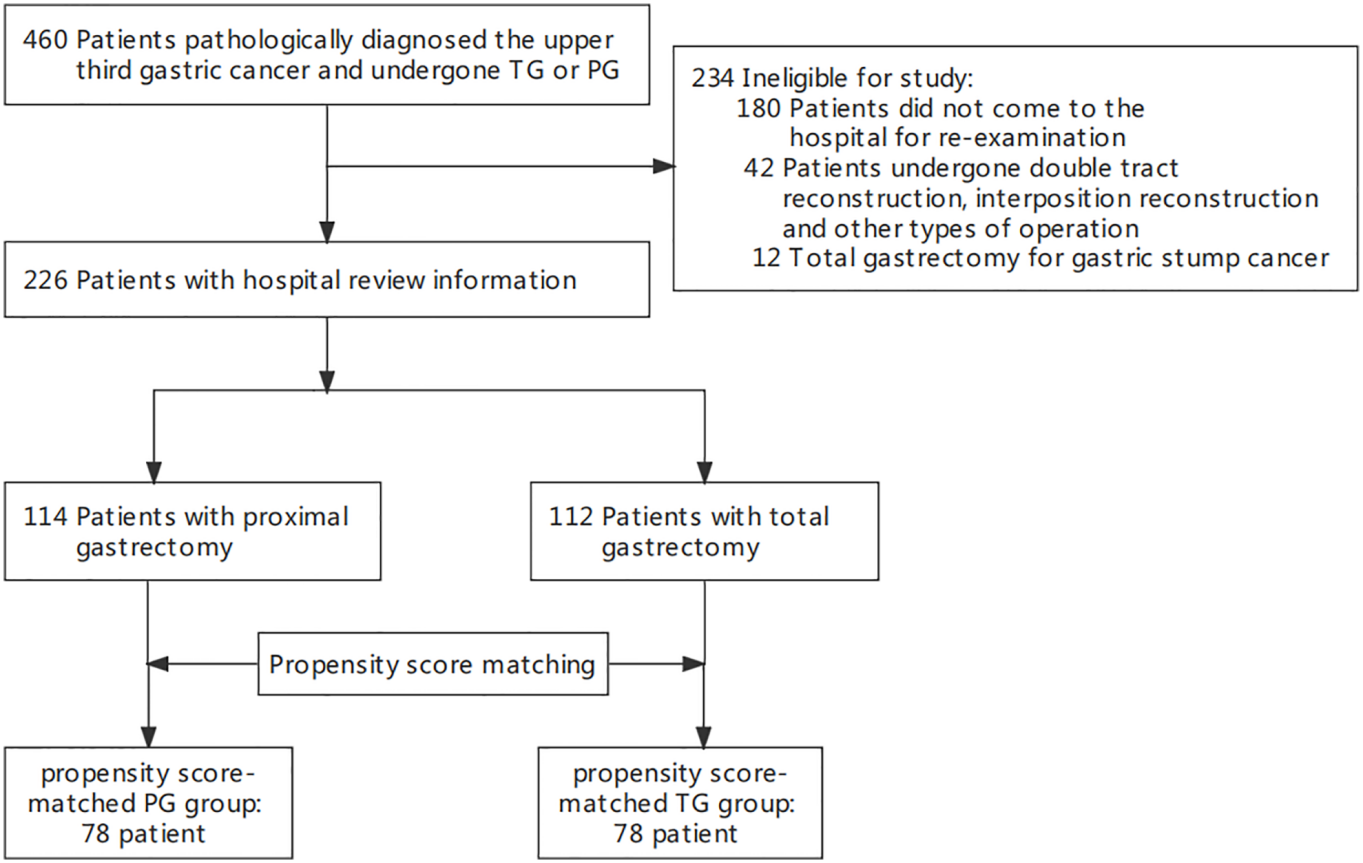
Exclusion and Inclusion Criteria.

**Figure 1** showed the study flowchart, 226 patients with EGJ cancer were finally included in the study: 114 PG group, and 112 TG group. The demographic and surgical characteristics were presented in **Table 1**. The mean (SD) age of the whole group was 58.9 (10.5) years old and 81.9% of them were male. No significant differences were observed between the two groups with respect to all the preoperative demographics background, like sex, age, BMI and preoperative comorbidities. Similarly, surgical approach, estimated blood loss, and length of hospital stay were also comparable between the two groups, but the total number of lymph node biopsied in the TG group was significantly higher than PG group (26.34% vs 14.58; p = 0.005) (**Table 1**). Of the postoperative histopathologic characteristics, tumor size, Lauren types, Borrmann types, Siewert classifications, Vessel invasion, Nerve invasion, T and N stages and pathologic tumor stage exhibited statistically significant differences between groups (**Table 2**). Curative resection (R0) was performed in all patients.

**Table 1:** Demographics and surgical characteristics of patients undergoing PG and TG.

	PG	TG	p
n	78	78	
**Sex**			0.519
male	63 (80.8)	67 (85.9)	
female	15 (19.2)	11 (14.1)	
**Age, mean (SD)**	59.77 (9.87)	58.87 (9.89)	0.571
**Age**			0.359
<65	55 (70.5)	61 (78.2)	
≥65	23 (29.5)	17 (21.8)	
**Disease**			0.413
No	44 (56.4)	50 (64.1)	
Yes	34 (43.6)	28 (35.9)	
**Height0**	168.40 (6.90)	168.95 (6.76)	0.615
**Weight0**	70.42 (13.38)	70.59 (12.15)	0.933
**BMI0**	24.78 (4.23)	24.65 (3.50)	0.84
**Surgical approach**			0.296
Laparotomy	58 (74.4)	53 (67.9)	
Laparoscopy	20 (25.6)	23 (29.5)	
Laparoscopy converted to Laparotomy	0 (0.0)	2 (2.6)	
**Blood loss (ml)**	138.67 (78.77)	150.38 (138.25)	0.672
**Lymph node biopsy**	32.10 (16.32)	37.53 (13.72)	0.107
**Length of hospital stay**	20.78 (12.15)	18.63 (6.82)	0.174
**R0 resection**	78 (100.0)	78 (100.0)	-

**Table 2:** Comparison of pathological characteristics.

	PG	TG	p
**Total**	78	78	
**Size, mean (SD)**	4.24 (1.88)	4.42 (1.96)	0.56
**Lauren type**			0.262
Intestinal	37 (47.4)	27 (34.6)	
Mixed	24 (30.8)	29 (37.2)	
Diffuse	17 (21.8)	22 (28.2)	
**Bormann type**			1
0-1	25 (32.1)	24 (30.8)	
2-4	53 (67.9)	73 (93.6)	
**Siewert classification**			<0.001
Siewert II	29 (37.2)	5 (6.4)	
Siewert III	49 (62.8)	54 (69.2)	
**Differentiation**			1
Poorly differentiated	59 (75.6)	58 (74.4)	
Well differentiated	19 (24.4)	20 (25.6)	
**Vessel invasion**			0.059
Negative	51 (67.1)	38 (50.7)	
Positive	25 (32.9)	37 (49.3)	
**Nerve invasion**			1
Negative	37 (48.1)	37 (47.4)	
Positive	40 (51.9)	41 (52.6)	
**Signet-ring cell**			0.136
No-Signet cells	62 (79.5)	64 (82.1)	
Partial-Signet cells	10 (12.8)	13 (16.7)	
Signet-ring cell carcinoma	6 (7.7)	1 (1.3)	
**Pathological T-stage**			0.25
T3-T4	57 (73.1)	64 (82.1)	
T1-T2	21 (26.9)	14 (17.9)	
**Pathological N-stage**			0.423
N0	41 (52.6)	35 (44.9)	
N0-N3	37 (47.4)	43 (55.1)	
**Pathological stage**			0.633
I	28 (35.9)	24 (30.8)	
II	22 (28.2)	26 (33.3)	
III	27 (34.6)	25 (32.1)	
IV	1 (1.3)	3 (3.8)	

### Short- and long-term outcomes

Postoperative short-term outcomes were presented in **Table 3**. The early postoperative complications in the PG group (26 patients, 23.0%) was higher than in the TG group (20 patients, 18.7%). The anastomotic stenosis in PG group was significantly higher than TG (6.1% vs 0.0%; p = 0.023), while there was no significant difference between the two groups as to any other short-term complications, such as anastomotic leakage, ileus, postoperative bleeding, surgical site infection and so on.

**Table 3:** Short-term outcomes.

	PG	TG	p
**n**	78	78	
**Death number**	27	30	
**Cause of death**			0.367
Tumor recurrence	22 (81.5)	26 (86.7)	
Intestinal rupture	2 (7.4)	0 (0.0)	
Myocardial infarction	0 (0.0)	1 (3.3)	
Pulmonary Fibrosis	1 (3.7)	0 (0.0)	
Traffic accident	0 (0.0)	1 (3.3)	
Other reasons	0 (0.0)	1 (3.3)	
Unknown	2 (7.4)	1 (3.3)	

Long-term complications (**Table 4**) were observed in 73 patients (64.0%) in the PG and 36 patients (32.1%) in the TG group, and the complication rate of PG was significantly higher than TG (p < 0.001). In the PG group, complications consisted of reflux esophagitis (n = 62, 54.4%), anastomotic stenosis (n = 17, 14.9%), ileus (n = 5, 4.4%) and ascites (n = 2, 1.8%). For the TG group, late complications included reflux esophagitis (n = 30, 26.8%), anastomotic stenosis (n = 5, 4.5%), ileus (n = 6, 5.4%) and ascites (n = 2, 1.8%).

**Table 4:** Long-term outcomes.

	PG	TG	p
**n**	114	112	
**complication rate**	73 (64.0)	36 (32.1)	<0.001
**Reflux esophagitis**	62 (54.4)	30 (26.8)	<0.001
**Severe esophagitis (LA classification C or D)**	35 (30.7)	16 (14.3)	0.005
**Visick grade**			<0.001
1	3 (2.6)	0 (0.0)	
2	44 (38.6)	23 (20.5)	
3	14 (12.3)	6 (5.4)	
4	1 (0.9)	0 (0.0)	
**Anastomotic stenosis**	17 (14.9)	5 (4.5)	0.015
**Ileus**	5 (4.4)	6 (5.4)	0.976
**Ascites**	2 (1.8)	2 (1.8)	1

Overall, the incidence of reflux esophagitis and anastomotic stenosis were significantly higher in the PG group than in the TG group (p = 0.001 and p=0.015, respectively). In addition, the grade C or D reflux esophagitis judged by the LA classification in PG group was significantly higher than TG (30.7% vs 14.3%, p = 0.005). Similarly, reflux esophagitis assessed by the Visick grade in PG group was also higher than TG group (p < 0.001). Besides, significant difference was also observed in the rates of anastomotic stenosis between the PG and TG groups (p = 0.015; 14.9% vs 4.5%).

### Postoperative nutritional status

**Figure 2:**
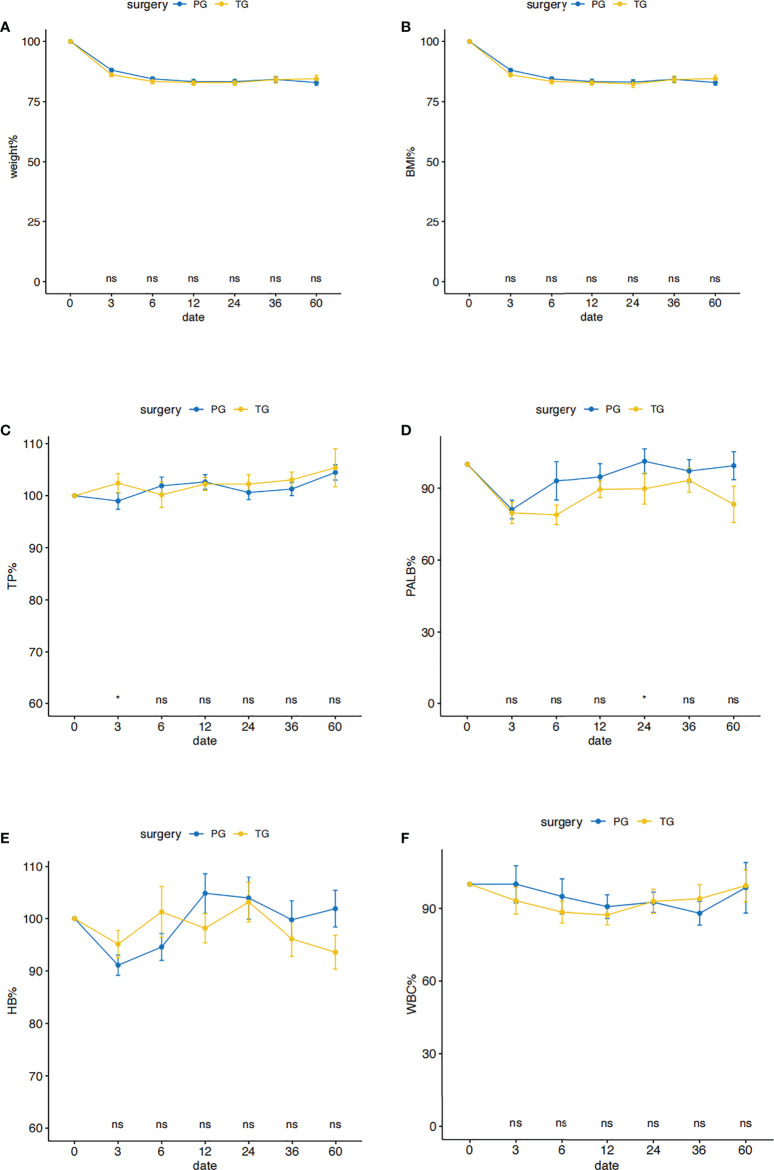
Postoperative nutritional status in the PG and the TG groups before PSM. Comparison of nutritional outcomes, Weight **(A)**, BMI **(B)**, TP **(C)**, PALB **(D)**, HB **(E)**, WBC **(F)** between PG and TG groups (TP: total protein, PALB: prealbumin, HB: hemoglobin, WBC: total leukocytes levels). All postoperative data are represented as a percentage of preoperative data. *p < 0.05: significance level. PG blue line, TG yellow line.

Postoperative %Weight and %BMI changes were shown in **Figures 2A, B**. There was no significant difference in the level of %Weight and %BMI changes at each time of follow-up between the two groups. The %Weight and %BMI changes of the two groups decreased rapidly within 3 months after surgery, and tended to be stable after 1 year, and then both increased slightly after 2 years. Transitions in postoperative total protein, prealbumin, hemoglobin and total leukocytes levels in the two groups were shown in **Figures 2C–F**, respectively. Overall, the nutritional indicators in both groups decreased in a short period of time after the operation, and then stabilized and reached the normal level as pre-operation. Total protein, hemoglobin and total leukocytes reduction rates were similar between the two groups at any time of examination in the whole period of 5 years after surgery, only except that at 3 months postoperatively, total protein percentage levels in the TG group were higher than the PG group (p < 0.05). Prealbumin in the PG group significantly higher than TG at the time of 2 years after surgery (p < 0.05), while it was similar between the two groups at the time of 5 years after surgery.

### Recurrence

Totally, 71 of 226(31.4%) patients relapsed after surgery. Among them, 24 were in the PG group and 47 were in the TG group, the difference was significant (P<0.05) (**Table 5**). Relapse in situ, regional lymph node, hematogenous, and dissemination recurrences developed in 5 (2.2%), 25(11.1%), 20 (8.8%), and 21 (9.3%) patients, respectively.

**Table 5:** A comparison of the recurrence between the PG and the TG groups.

	Overall	PG	TG	p
**n**		114	112	
**Recurrence rate**				0.002
Yes	70 (31.4)	24 (21.1)	47 (42.0)	
No	156 (68.6)	90 (78.9)	65 (58.0)	
**Recurrence site**				0.002
Local	5 (2.2)	4 (3.5)	1 (0.9)	
lymph node	25 (11.1)	9 (7.9)	16 (14.3)	
hematogenous	20 (8.8)	5 (4.4)	15 (13.4)	
dissemination	21 (9.3)	6 (5.3)	15 (13.4)	

### Survival outcomes after PSM analysis

**Figure 3:**
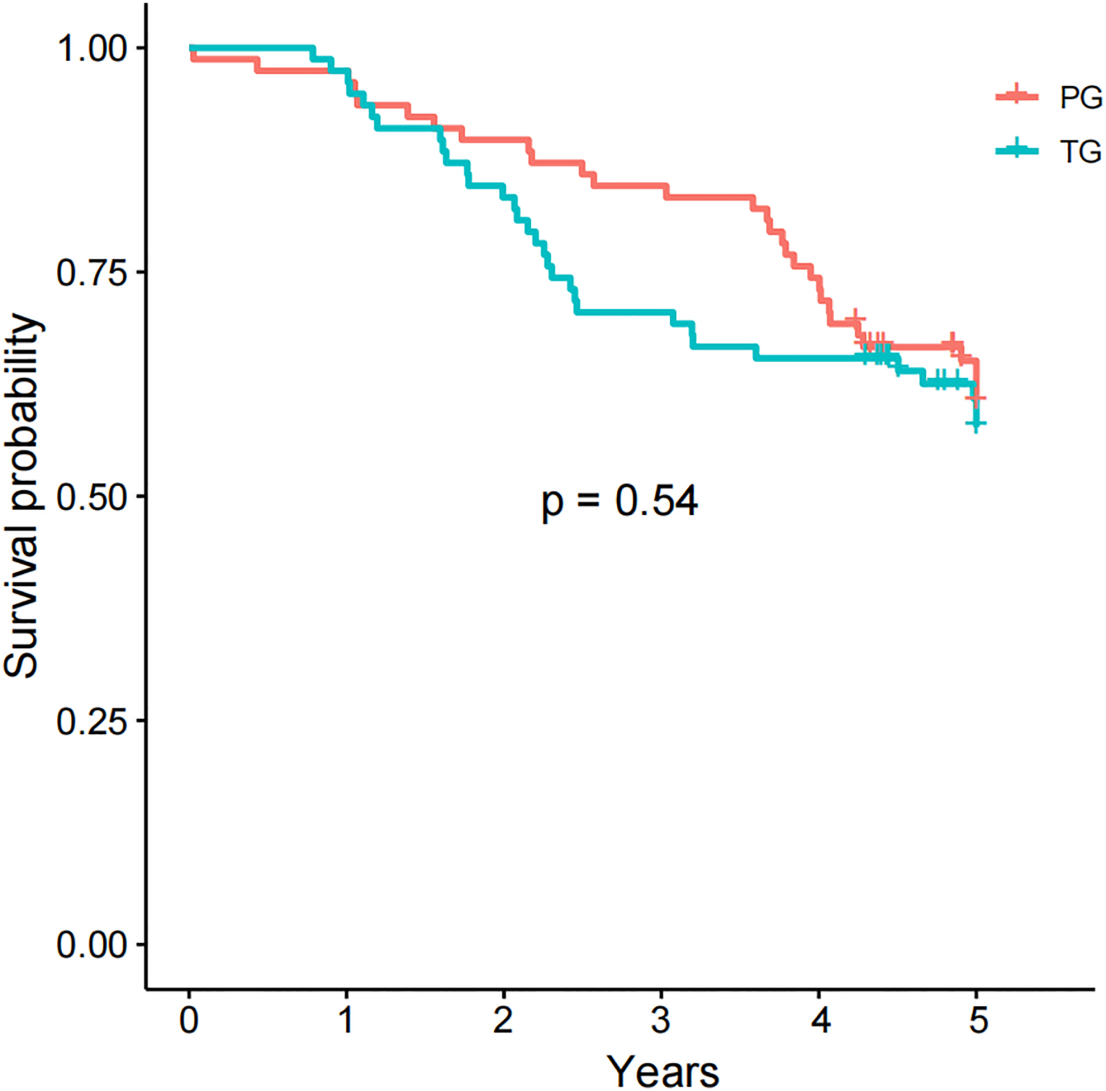
5-year overall survival in the PG and TG groups after PSM.

Finally, 78 people in the PG group and 78 people in the TG group were matched by PSM (**Supplementary Tables 1**, **2**). The 5-years overall survival in PG group was comparable to TG (65.4% versus 61.5%, respectively; p = 0.54, **Figure 3**). Besides, the causes of death in PG group were also similar to TG (**Supplementary Table 3**).

## Discussion

As reported, the incidence of EGJ cancer increased in recent decades (18, 19). For the treatment of EGJ cancer, TG is the mostly used procedure, both in early-stage and locally advanced gastric cancer patients (18, 20). Meanwhile, PG, a function‐preserving procedure, is advocated for lesions diagnosed at an early stage when more than half of the distal stomach can be preserved to maintain the nutritional status of patients (18, 19), and now more and more surgeons tend to perform PG for advanced proximal gastric cancer (7, 21). However, the severe postoperative reflux and anastomotic stenosis make the quality of life of many patients poor (13, 22), and what’s more there is a risk of developing gastric stump cancer (GSC) after PG with partial stomach preserved (23, 24). Several studies have reported controversial results concerning the nutritional status of patients underwent PG or TG, and nearly all of the studies focused in the first 2 years after surgery. So we conducted this study to compare the long-term nutritional outcomes and found they were similar at the time of 5 years after surgery.

Postoperative %BMI is the most important indicator of postoperative nutritional status because postoperative malnutrition is one of the most common disturbs experienced by patients undergoing gastrectomy. In our study, we found that %BMI were basically similar in the PG and TG groups as a long-term result. Similarly, Cho et al. (9) also found that %BMI changes from baseline were not significantly different between PG group and TG group in first 2 years after surgery. More aggressively, An et al. (10) condemned that PG provided no benefit in terms of postoperative %BMI changes and was associated with a markedly higher rate of long-term postoperative complications.

Furthermore, there were no differences among the two treatment groups in our study in terms of postoperative nutritional transitions, consistent with previous reports of EGJ cancer, which reported that nutritional status indicators including hemoglobin, total protein, and total leukocyte count for EGJ cancer, were not significantly different between PG group and TG group within three years after surgery (9, 14). Besides, Cho et al. (9) argued that Patients in both groups experienced a similar cumulative incidence of iron deficiency anemia, which was almost identical during a median follow-up period of 24 months postoperatively (P = 0.971). Overall, our results for %BMI and nutritional status were in line with these results.

However, a few reports have reported that TG resulted in worse postoperative %BMI changes and long-term nutritional status than PG (12, 25, 26). Yamasaki et al. (12) found that the long-term nutritional status including %BMI, %Weight, some hematological tests, were significantly higher in patients who underwent PG than TG for the EGJ cancer. Given these results, surgical procedure may not be a decisive factor for postoperative malnutrition in EGJ patients.

For postoperative complication assessment, some retrospective studies have reported that the incidence of anastomotic stenosis and reflux esophagitis in the PG group was significantly higher than that in the TG group (10, 11, 13, 22), which was also in agree with the results of the present study. An et al. (10) found that PG was associated with a markedly higher rate of anastomotic stenosis and reflux esophagitis. The results of Kim et al. (22) showed that the rates of anastomosis stenosis and reflux esophagitis were 46.5 and 48% respectively after PG, and it’s really high. Although some new methods of digestive tract reconstruction after PG, such as jejunal interposition (JI), and double-tract reconstruction (ET), Kamikaw, and overlap so on, were developed, the long-term benefit still needed to be confirmed.

In the present study, overall postoperative survival rates were comparable between the PG and TG groups after PSM, while TG used to be thought as the standard surgical method of advanced EGJ cancer for better survival and lower recurrence (18, 22, 27) The possible reason for the similar survival of PG and TG might be that the patients who received PG had a tumor size smaller than 4cm and no obvious enlarged lymph anode. However, patients who underwent PG would inevitably have the risk of cancer of remnant stomach as high as 5.4%.

The present study has several limitations. Firstly, the analysis was based on retrospective data collected at a single institution and included a relatively small number of patients. Due to the retrospective nature of this study, a selection bias existed between the PG and TG groups. While the selection bias in this study might not be so important as other studies focused on survival, the present study concerned on those who survived more than 5 years. Secondly, only the Visick symptom grade and LA classification were used for grading postoperative esophageal reflux. In fortunately, all of the patients received endoscopy examination every year after surgery, and the diagnosis of reflux esophagitis was credible. Finally, although the reconstruction methods of digestive tract included several kinds such as traditional esophageal-gastrostomy, JI, ET and overlap, traditional method took the predominant, and the study endpoint was still comparative between PG and TG.

In conclusion, TG should be more aggressively recommended for the similar nutritional status, significantly lower reflux esophagitis and anastomotic stenosis, and free of carcinoma of remnant stomach compared with PG.

## Data availability statement

The raw data supporting the conclusions of this article will be made available by the authors, without undue reservation.

## Ethics statement

Written informed consent was obtained from the individual(s) for the publication of any potentially identifiable images or data included in this article.

## Author contributions

SD and XZ contributed to study conception and manuscript writing and data analysis. SD, XZ, SW, MW, YW, and CS contributed to data collection. YX, CW, SW, MW and CS contributed to clinical treatment and diagnosis. All authors contributed to the article and approved the submitted version.

## Funding

This work was supported by CAMS Innovation Fund for Medical Sciences (CIFMS No. 2016-I2M-1-001 and No. 2016-I2M-1-007) and National Natural Science Foundation of China (81972314).

## Conflict of interest

The authors declare that the research was conducted in the absence of any commercial or financial relationships that could be construed as a potential conflict of interest.

## Publisher’s note

All claims expressed in this article are solely those of the authors and do not necessarily represent those of their affiliated organizations, or those of the publisher, the editors and the reviewers. Any product that may be evaluated in this article, or claim that may be made by its manufacturer, is not guaranteed or endorsed by the publisher.
