# Supplementary material for: Long-term nutritional status after total gastrectomy was comparable to proximal gastrectomy but with much less reflux esophagitis and anastomotic stenosis
Source: Front Oncol. 2022 Oct 25;12:973902. doi: 10.3389/fonc.2022.973902 (PMC9641152; doi:10.3389/fonc.2022.973902)
Supplement: Supplementary file 1 [file Table_1.docx]

**Supplementary Table 1 Demographics and surgical characteristics of patients undergoing PG and TG after PSM**

|  | **PG** | **TG** | **p** |
| --- | --- | --- | --- |
| n | 78 | 78 |  |
| **Sex** |  |  | 0.519 |
| male | 63 (80.8) | 67 (85.9) |  |
| female | 15 (19.2) | 11 (14.1) |  |
| **Age, mean (SD)** | 59.77 (9.87) | 58.87 (9.89) | 0.571 |
| **Age** |  |  | 0.359 |
| <65 | 55 (70.5) | 61 (78.2) |  |
| ≥65 | 23 (29.5) | 17 (21.8) |  |
| **Disease** |  |  | 0.413 |
| No | 44 (56.4) | 50 (64.1) |  |
| Yes | 34 (43.6) | 28 (35.9) |  |
| **Height0** | 168.40 (6.90) | 168.95 (6.76) | 0.615 |
| **Weight0** | 70.42 (13.38) | 70.59 (12.15) | 0.933 |
| **BMI0** | 24.78 (4.23) | 24.65 (3.50) | 0.84 |
| **Surgical approach** |  |  | 0.296 |
| Laparotomy | 58 (74.4) | 53 (67.9) |  |
| Laparoscopy | 20 (25.6) | 23 (29.5) |  |
| Laparoscopy converted to Laparotomy | 0 (0.0) | 2 (2.6) |  |
| **Blood loss (ml)** | 138.67 (78.77) | 150.38 (138.25) | 0.672 |
| **Lymph node biopsy** | 32.10 (16.32) | 37.53 (13.72) | 0.107 |
| **Length of hospital stay** | 20.78 (12.15) | 18.63 (6.82) | 0.174 |
| **R0 resection** | 78 (100.0) | 78 (100.0) | - |
