# Supplementary material for: Long-term nutritional status after total gastrectomy was comparable to proximal gastrectomy but with much less reflux esophagitis and anastomotic stenosis
Source: Front Oncol. 2022 Oct 25;12:973902. doi: 10.3389/fonc.2022.973902 (PMC9641152; doi:10.3389/fonc.2022.973902)
Supplement: Supplementary file 2 [file Table_2.docx]

**Supplementary Table 2 Comparison of pathological characteristics after PSM**

|  | **PG** | **TG** | **p** |
| --- | --- | --- | --- |
| **Total** | 78 | 78 |  |
| **Size, mean (SD)** | 4.24 (1.88) | 4.42 (1.96) | 0.56 |
| **Lauren type** |  |  | 0.262 |
| Intestinal | 37 (47.4) | 27 (34.6) |  |
| Mixed | 24 (30.8) | 29 (37.2) |  |
| Diffuse | 17 (21.8) | 22 (28.2) |  |
| **Bormann type** |  |  | 1 |
| 0-1 | 25 (32.1) | 24 (30.8) |  |
| 2-4 | 53 (67.9) | 73 (93.6) |  |
| **Siewert classification** |  |  | <0.001 |
| Siewert II | 29 (37.2) | 5 (6.4) |  |
| Siewert III | 49 (62.8) | 54 (69.2) |  |
| **Differentiation** |  |  | 1 |
| Poorly differentiated | 59 (75.6) | 58 (74.4) |  |
| Well differentiated | 19 (24.4) | 20 (25.6) |  |
| **Vessel invasion** |  |  | 0.059 |
| Negative | 51 (67.1) | 38 (50.7) |  |
| Positive | 25 (32.9) | 37 (49.3) |  |
| **Nerve invasion** |  |  | 1 |
| Negative | 37 (48.1) | 37 (47.4) |  |
| Positive | 40 (51.9) | 41 (52.6) |  |
| **Signet-ring cell** |  |  | 0.136 |
| No-Signet cells | 62 (79.5) | 64 (82.1) |  |
| Partial-Signet cells | 10 (12.8) | 13 (16.7) |  |
| Signet-ring cell carcinoma | 6 (7.7) | 1 (1.3) |  |
| **Pathological T-stage** |  |  | 0.25 |
| T3-T4 | 57 (73.1) | 64 (82.1) |  |
| T1-T2 | 21 (26.9) | 14 (17.9) |  |
| **Pathological N-stage** |  |  | 0.423 |
| N0 | 41 (52.6) | 35 (44.9) |  |
| N0-N3 | 37 (47.4) | 43 (55.1) |  |
| **Pathological stage** |  |  | 0.633 |
| I | 28 (35.9) | 24 (30.8) |  |
| II | 22 (28.2) | 26 (33.3) |  |
| III | 27 (34.6) | 25 (32.1) |  |
| IV | 1 (1.3) | 3 (3.8) |  |
