# Supplementary material for: Long-term nutritional status after total gastrectomy was comparable to proximal gastrectomy but with much less reflux esophagitis and anastomotic stenosis
Source: Front Oncol. 2022 Oct 25;12:973902. doi: 10.3389/fonc.2022.973902 (PMC9641152; doi:10.3389/fonc.2022.973902)
Supplement: Supplementary file 3 [file Table_3.docx]

**Supplementary Table 3 Cause of death of deceased patient after PSM**

|  | **PG** | **TG** | **p** |
| --- | --- | --- | --- |
| **n** | 78 | 78 |  |
| **Death number** | 27 | 30 |  |
| **Cause of death** |  |  | 0.367 |
| Tumor recurrence | 22 (81.5) | 26 (86.7) |  |
| Intestinal rupture | 2 (7.4) | 0 (0.0) |  |
| Myocardial infarction | 0 (0.0) | 1 (3.3) |  |
| Pulmonary Fibrosis | 1 (3.7) | 0 (0.0) |  |
| Traffic accident | 0 (0.0) | 1 (3.3) |  |
| Other reasons | 0 (0.0) | 1 (3.3) |  |
| Unknown | 2 (7.4) | 1 (3.3) |  |
